# Multicriteria Optimization of Language Models for Heart Failure With Preserved Ejection Fraction Symptom Detection in Spanish Electronic Health Records: Comparative Modeling Study

**DOI:** 10.2196/76433

**Published:** 2025-07-17

**Authors:** Jacinto Mata, Victoria Pachón, Ana Manovel, Manuel J Maña, Manuel de la Villa

**Affiliations:** 1 I²C Research Group, Universidad de Huelva, Huelva, 21007, Spain, +34 687862089; 2 Cardiology Department, Juan Ramón Jiménez University Hospital, Multidisciplinary Amyloidosis Unit Huelva, Hospital Juan Ramón Jiménez, Huelva, Spain

**Keywords:** natural language processing, transformer, clinical language models, manual corpus annotation, symptom extraction, early diagnosis support

## Abstract

**Background:**

Heart failure with preserved ejection fraction (HFpEF) is a major clinical manifestation of cardiac amyloidosis, a condition frequently underdiagnosed due to its nonspecific symptomatology. Electronic health records (EHRs) offer a promising avenue for supporting early symptom detection through natural language processing. However, identifying relevant clinical cues within unstructured narratives, particularly in Spanish, remains a significant challenge due to the scarcity of annotated corpora and domain-specific models. This study proposes and evaluates a Transformer-based natural language processing framework for automated detection of HFpEF-related symptoms in Spanish EHRs.

**Objective:**

The aim of this study is to assess the feasibility of leveraging unstructured clinical narratives to support early identification of heart failure phenotypes indicative of cardiac amyloidosis. It also examines how domain-specific language models and clinically guided optimization strategies can improve the reliability, sensitivity, and generalizability of symptom detection in real-world EHRs.

**Methods:**

A novel corpus of 15,304 Spanish clinical documents was manually annotated and validated by cardiology experts. The corpus was derived from the records of 262 patients (173 with suspected cardiac amyloidosis and 89 without). In total, 8 Transformer-based language models were evaluated, including general-purpose models, biomedical-specialized variants, and Longformers. Three clinically motivated optimization strategies were implemented to align models’ behavior with different diagnostic priorities: maximizing area under the curve (AUC) to enhance overall discrimination, optimizing *F*_1_-score to balance sensitivity and precision, and prioritizing sensitivity to minimize false negatives. These strategies were independently applied during the fine-tuning of the models to assess their impact on performance under different clinical constraints. To ensure robust evaluation, testing was conducted on a dataset composed exclusively of previously unseen patients, allowing performance to be assessed under realistic and generalizable conditions.

**Results:**

All models achieved high performance, with AUC values above 0.940. The best-performing model, *Longformer Biomedical-clinical*, reached an AUC of 0.987, *F*_1_-score of 0.985, sensitivity of 0.987, and specificity of 0.987 on the test dataset. Models optimized for sensitivity reduced the false-negative rate to under 3%, a key threshold for clinical safety. Comparative analyses confirmed that domain-adapted, long-sequence models are better suited for the semantic and structural complexity of Spanish clinical texts than general-purpose models.

**Conclusions:**

Transformer-based models can reliably detect HFpEF-related symptoms from Spanish EHRs, even in the presence of class imbalance and substantial linguistic complexity. The results show that combining domain-specific pretraining with long-context modeling architectures and clinically aligned optimization strategies leads to substantial gains in classification performance, particularly in sensitivity. These models not only achieve high accuracy and generalization on unseen patients but also demonstrate robustness in handling the semantic nuances and narrative structure of real-world clinical documentation. These findings support the potential deployment of Transformer-based systems as effective screening tools to prioritize patients at risk for cardiac amyloidosis in Spanish-speaking health care settings.

## Introduction

### Background

Heart failure (HF) represents a major public health challenge, with its prevalence rising as a result of population aging and improved survival among individuals with cardiovascular conditions. Among its phenotypes, heart failure with preserved ejection fraction (HFpEF) accounts for nearly 50% of cases and is characterized by congestion and dyspnea in the absence of marked systolic dysfunction. Despite its clinical relevance, HFpEF remains diagnostically challenging due to its pathophysiological and etiological heterogeneity [1].

A key underlying cause of HFpEF is cardiac amyloidosis, an infiltrative disorder characterized by extracellular deposits of misfolded transthyretin protein that impair myocardial structure and function. The most prevalent variant is the wild type form of transthyretin cardiac amyloidosis (ATTR-CM), which predominantly affects older adults and leads to progressive cardiac dysfunction and worsening HF symptoms. Unlike other HFpEF etiologies, ATTR-CM is amenable to disease-modifying therapies that can slow progression, reduce hospitalizations, and improve survival outcomes. Nonetheless, early diagnosis remains difficult due to low clinical suspicion, nonspecific echocardiographic findings, and historically limited awareness within the medical community. Therapeutic efficacy is maximized when treatment is initiated early, underscoring the importance of timely and optimized detection strategies in HF populations [2].

In this context, artificial intelligence (AI) has emerged as a promising approach for the early identification of disease within large-scale clinical datasets [3]. Specifically, natural language processing (NLP) and deep learning models can analyze electronic health records (EHRs) to identify and extract mentions of HFpEF and associated terminology, facilitating the development of high-quality annotated corpora [4]. Such corpora are essential for training AI models capable of detecting textual patterns indicative of ATTR-CM, enabling the prioritization of high-risk patients. Applying these technologies to the structured identification of HF mentions in clinical narratives can enhance diagnostic accuracy, support data-driven decision-making, and enable timely interventions that improve prognosis and quality of life.

Despite the growing interest in clinical NLP, the development of resources in Spanish remains limited. The scarcity of expert-annotated clinical corpora in Spanish hinders the creation and evaluation of language models tailored to this language. The lack of pretrained clinical language models in Spanish, compared to the resources available for English, further restricts the development of effective applications in this domain. Recently, García Subies et al [5] conducted a study on the efficiency of encoder-based Transformer models in named entity recognition and classification tasks, aiming to identify the most effective resources in this context. The authors highlighted the significant gap in NLP resources for the Spanish language, particularly in the clinical sector. As described in their work, annotated corpora in Spanish are extremely scarce, and most are designed for named entity recognition tasks, with very few available for text classification. The authors noted that, when working with clinical narratives, encoder-based models currently outperform emerging generative language models. Their findings emphasize the urgent need to develop encoder-based models specialized in Spanish that can process clinical data with high precision. One of the most significant challenges in clinical NLP, both in general and particularly in Spanish, is the handling of negation and speculation in clinical texts. Although some initiatives such as *NUBes* [6] have emerged, available resources to properly address this linguistic feature remain extremely limited. As a result, this remains one of the most relevant and complex issues currently faced by the scientific community in the field.

Our study aims to develop and validate an AI-based system for the automated detection of HFpEF mentions in Spanish EHRs, with the goals of supporting early detection of potential ATTR-CM cases, enhancing diagnostic efficiency, and mitigating underdiagnosis. As outlined in [7], several research challenges remain in clinical NLP. This work addresses three of them: applying deep learning to clinical text classification, overcoming language-related barriers, and leveraging transfer learning for narrative clinical report classification.

To guide this work, we pose the following research questions (RQs):

RQ1. Can a manually annotated corpus of Spanish EHRs be effectively constructed and validated for the detection of HFpEF-related symptoms?

RQ2. What is the performance of general-purpose, biomedical-pretrained, and long-document, encoder-based Transformer models when applied to clinical text classification in Spanish?

RQ3. What is the impact of different optimization strategies (area under the curve [AUC], *F*_1_-score, and sensitivity) on model performance for symptom detection?

RQ4. To what extent can encoder-based Transformer models support the early identification of HFpEF symptomatology indicative of cardiac amyloidosis in Spanish-language clinical narratives?

To address these questions, this study makes the following contributions: (1) the construction of a manually annotated corpus of Spanish EHRs for HFpEF symptom detection, validated by cardiology specialists and intended for public release; (2) a comparative evaluation of general, biomedical, and long-document Transformer models for clinical text classification; (3) an analysis of optimization strategies tailored to AUC, *F*_1_-score, and sensitivity; and (4) a demonstration of the feasibility of using NLP models to support the early detection of HFpEF symptoms relevant to cardiac amyloidosis.

### Related Work

Transformer-based models have shown increasing effectiveness in extracting clinically relevant information from unstructured EHRs, particularly within the context of HF. Adejumo et al [8] used ClinicalBERT to extract New York Heart Association classifications and HF symptoms from clinical notes, achieving area under the receiver operating characteristic curves (AUROCs) greater than 0.98 and identifying functional status in 83% more cases than explicit documentation alone. Similarly, Liu et al [9] used ClinicalBERT embeddings within a predictive framework to identify early HF onset, outperforming traditional methods in a large-scale real-world dataset.

In the context of clinical trials, Marti-Castellote et al [10] developed a hybrid NLP approach integrating Clinical Longformer and GPT-4o to adjudicate HF-related hospitalizations. Their system reproduced expert adjudications with 83% concordance, substantially reducing manual effort and demonstrating the scalability and reliability of Transformer-based models in high-stakes research. Ahmad et al [11] provided a comprehensive overview of machine learning applications in HFpEF, emphasizing the potential of unsupervised learning techniques to identify novel patient subgroups and improve phenotypic classification. Their work underscores the importance of leveraging diverse data modalities, including clinical notes, to enhance diagnostic precision in HFpEF. Complementarily, Houssein et al [12] developed an advanced NLP framework using stacked BERT and character embeddings to detect heart disease risk factors from clinical narratives. Their model achieved an *F*_1_-score of 93.66% on the i2b2 dataset, demonstrating the efficacy of combining deep learning techniques for extracting critical clinical information. Houssein et al [13] benchmarked 5 Transformer architectures (BERT, BioBERT, RoBERTa, XLNet, and BioClinicalBERT) on the i2b2 dataset for risk factor extraction in heart disease, achieving state-of-the-art performance with a micro *F*_1_-score of 94.26%. These results reinforce the value of domain-specific Transformer models in clinical NLP applications. Notably, Fan et al [14] implemented a Transformer-based clustering framework to identify 7 HF subtypes in a cohort of more than 379,000 patients. These subgroups, some of which were independent of left ventricular ejection fraction, highlight the capacity of deep learning approaches to uncover novel and clinically meaningful HF phenotypes, which is particularly relevant given the heterogeneity of HFpEF. These studies highlight the growing role of machine learning and NLP in advancing HF research and patient care. In the context of the Spanish language, recent work such as that of García Subies et al [5] has explored the use of encoder-based Transformer models for named entity recognition and classification tasks in Spanish clinical texts. However, their efforts have focused on the evaluation and application of language models on existing corpora and have not addressed the detection of HFpEF-specific symptoms.

Despite these advances, most of the existing research focuses on English-language EHRs, frequently relying on structured data or datasets with broad cardiovascular end points. To date, only a limited number of studies have addressed the automated detection of HFpEF-specific symptoms and none, to our knowledge, have done so in Spanish-language clinical narratives. Moreover, existing models are rarely optimized for clinically critical metrics such as sensitivity, nor do they account for the long and complex structure of real-world clinical narratives. This study addresses these gaps by introducing a manually annotated Spanish-language corpus for HFpEF symptom detection, evaluating multiple Transformer architectures, including models adapted for long documents, and implementing task-specific optimization strategies aligned with diagnostic priorities. This represents the first demonstration of the feasibility and clinical applicability of Transformer-based NLP for detecting HFpEF in Spanish EHRs, with direct implications for improving early recognition of cardiac amyloidosis.

## Methods

### Ethical Considerations

This study was approved by the Andalusian Biomedical Research Ethics Coordinating Committee under protocol version 1 dated October 2, 2020 (internal protocol code 2382-N-20). The corpus documents were anonymized using a rigorous, tailor-made protocol specifically designed to protect patient data privacy.

### The Corpus

#### Overview

Cardiac amyloidosis may present with a broad spectrum of clinical manifestations. This study focuses on HFpEF, one of the most clinically significant manifestations, to evaluate the performance of language models in detecting its presence or absence within clinical documents. Accordingly, the experiments have been conducted using a dataset specifically annotated for HFpEF. The full corpus, comprising 15 datasets of manually annotated clinical documents covering all cardiac amyloidosis symptoms, will be introduced in future publications. This corpus will be made publicly available to the scientific community.

#### Description

The corpus was compiled from the EHRs of 262 patients. The records of 173 patients who underwent testing for cardiac amyloidosis were selected and supplemented with the records of 89 patients who did not undergo these tests, aiming to mitigate overfitting in the models. These records were obtained from the Cardiology, Internal Medicine, Neurology, Neurophysiology, and Traumatology units at Juan Ramón Jiménez Hospital in Huelva, Spain. The selection of documents was based on patient records rather than predefined time intervals. Consequently, the temporal coverage of the dataset corresponds to the full range of available clinical documentation for each patient. Specifically, the corpus spans from 2007 to 2021, reflecting the period during which these patients received care and generated clinical records within the EHR system.

Following a detailed analysis to determine the most relevant document types for this study, the following clinical records were selected: *anamnesis*, *consultation reports*, *discharge reports*, and *progress notes*. To accommodate language model context limitations, documents were segmented into clinically relevant sections: *current illness*, *clinical assessment*, *reason for consultation*, *medical history*, and *complementary tests*. For progress notes, only the *evolution*, *clinical assessment*, and *complementary test* sections were retained to prioritize relevant content and reduce redundancy.

Table 1 provides the total number of documents per type, along with the overall corpus size. A data cleaning process was performed to eliminate duplicate documents, system-generated records without clinical content, and incomplete texts (eg, entries with minimal or blank content). Additionally, documents exhibiting a high degree of textual overlap were removed. This step was necessary because in many cases, clinical documents are generated as continuations of previous notes, incorporating incremental updates (eg, results of new tests or follow-up visits). To avoid excessive redundancy and reduce the risk of overfitting due to repetitive content, we retained only documents corresponding to distinct clinical episodes. When multiple documents were associated with the same episode, we preserved the longest version, as it typically contained the most comprehensive clinical information. Minor extraction errors were also corrected. As a result of this process, the corpus was reduced from 30,367 to 15,304 documents. Of these, 11,025 (72%) correspond to patients with suspected cardiac amyloidosis (n=173), and 4279 (28%) to control patients without the disease (n=89).

Table 2 presents summary statistics of document length by type, providing an initial overview of the textual characteristics that motivate the modeling approach adopted in this study. The documents in the corpus show considerable variability in length, with average values ranging from approximately 60 to over 120 words across document types. In all cases, the mean values exceed the median, suggesting a positively skewed distribution characterized by the presence of a relevant subset of longer documents that contribute to an extended upper tail. This is further supported by high standard deviation values and maximum word counts exceeding 2000 in some cases. These characteristics underscore the need for using long-context Transformer models capable of handling extended sequences without truncation, as will be demonstrated in the experimentation and results sections of this study. A sample of the dataset is presented in Multimedia Appendix 1 .

**Table 1.** Number of documents by type.

Document type	Number of documents
Anamnesis	5040
Consultation reports	5235
Discharge reports	3970
Progress notes	16,122
Total	30,367
Total after cleaning process	15,304

**Table 2.** Summary statistics of document length (in words) by document type. For each type of clinical note included in the corpus, the table presents the mean, median, standard deviation, and maximum number of words per document. The values reflect the variability in structure and verbosity across clinical document genres.

Document type	Mean (SD)	Median	Maximum
Anamnesis	90.12 (139.3)	40	1581
Consultation reports	124.41 (156.45)	62	1228
Discharge reports	116.59 (172.04)	52	2253
Progress notes	62.93 (75.13)	37	852

#### Labeling Process

This section outlines the corpus labeling process, with particular emphasis on the collection related to HFpEF, as this dataset was used for the experiments presented in this study. The annotation process followed a rigorous methodology appropriate for this type of task. Given the dataset’s size, its manual annotation required 6 months, highlighting the complexity of this phase of the study.

**Figure 1.**
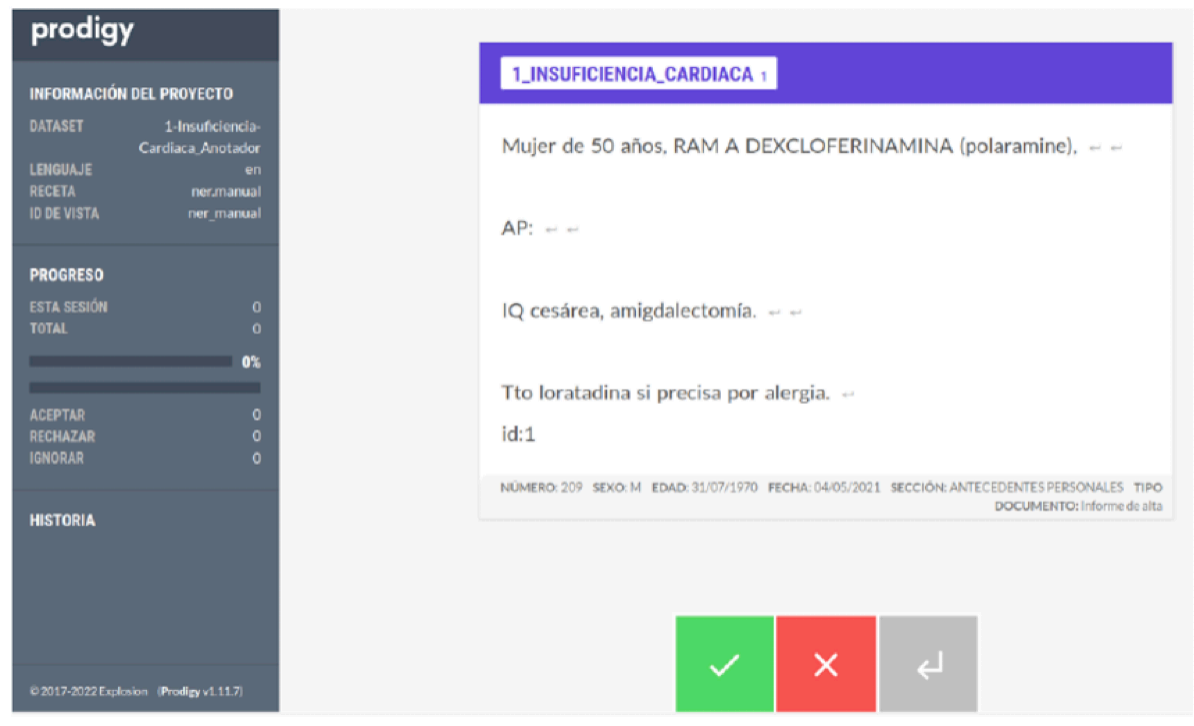
Prodigy interface for dataset annotation.

Based on prior evaluations of annotation tools [15], Prodigy [16] was selected due to its adaptability to the project’s requirements and its ease of use. To enhance the annotators’ efficiency, improvements were made to the tool’s functionality, and a user-friendly interface was designed to streamline the annotation process. Figure 1 illustrates the interface developed for this task.

To ensure consistency and standardization in the annotation process, an annotation guideline was developed under the supervision of a cardiology specialist. This guideline provides a detailed framework on relevant linguistic elements (terms, acronyms, and expressions, among others) that annotators must consider in determining the presence (positive case) or absence (negative case) of the symptom in the analyzed documents. Table 3 presents an excerpt from the annotation guide, illustrating some of the expressions used to indicate HF. Manual labeling was carried out by 2 specialists in medical documentation. To minimize annotation bias, the task was performed independently by each annotator, without mutual interaction. Discrepancies were resolved by the cardiology specialist who authored the annotation guidelines, ensuring consistency and validity.

**Table 3.** Excerpt from the annotation guide with some of the expressions used to label the dataset (English translation is included to facilitate reading).

Language and expressions indicating the presence of the symptom		Expressions indicating the absence of the symptom
**Spanish**		
	*Ingreso por ICC*^a^ *con FEVI*^b^ *preservada*	*Sin signos de IC* ^c^
	*I Cardiaca con FE*^d^ *conservada*	*ICC sin especificar si preservada o reducida*
	*Insuficiencia cardiaca diastólica*	*Insuficiencia cardiaca con FEVI reducida*
	*IC con función sistólica conservada o preservada*	*IC con FE disminuida*
	*CF*^e^ *III*	*IC sistólica*
	*EAP* ^f^	
	*Edema agudo de pulmón*	
	NYHA^g^ III	
**English**		
	Admitted for CHF^h^ with preserved LVEF^i^	No signs of HF^j^
	Heart failure with preserved EF^k^	CHF unspecified if preserved or reduced
	Diastolic heart failure	Heart failure with reduced LVEF
	HF with preserved or conserved systolic function	HF with decreased EF
	Class III	Systolic HF
	APE^l^	
	Acute pulmonary edema	
	NYHA III	

a ICC: *insuficiencia cardiaca congestiva*.

b FEVI: *fracción de eyección del ventrículo izquierdo*.

c IC: *insuficiencia cardiaca*.

d FE: *fracción de eyección*.

e CF: *clase funcional*.

f EAP: *edema agudo pulmonar*.

g NYHA: New York Heart Association.

h CHF: congestive heart failure.

i LVEF: left ventricular ejection fraction.

j HF: heart failure.

k EF: ejection fraction.

l APE: acute pulmonary edema.

#### Annotation Statistics

**Figure 2.**
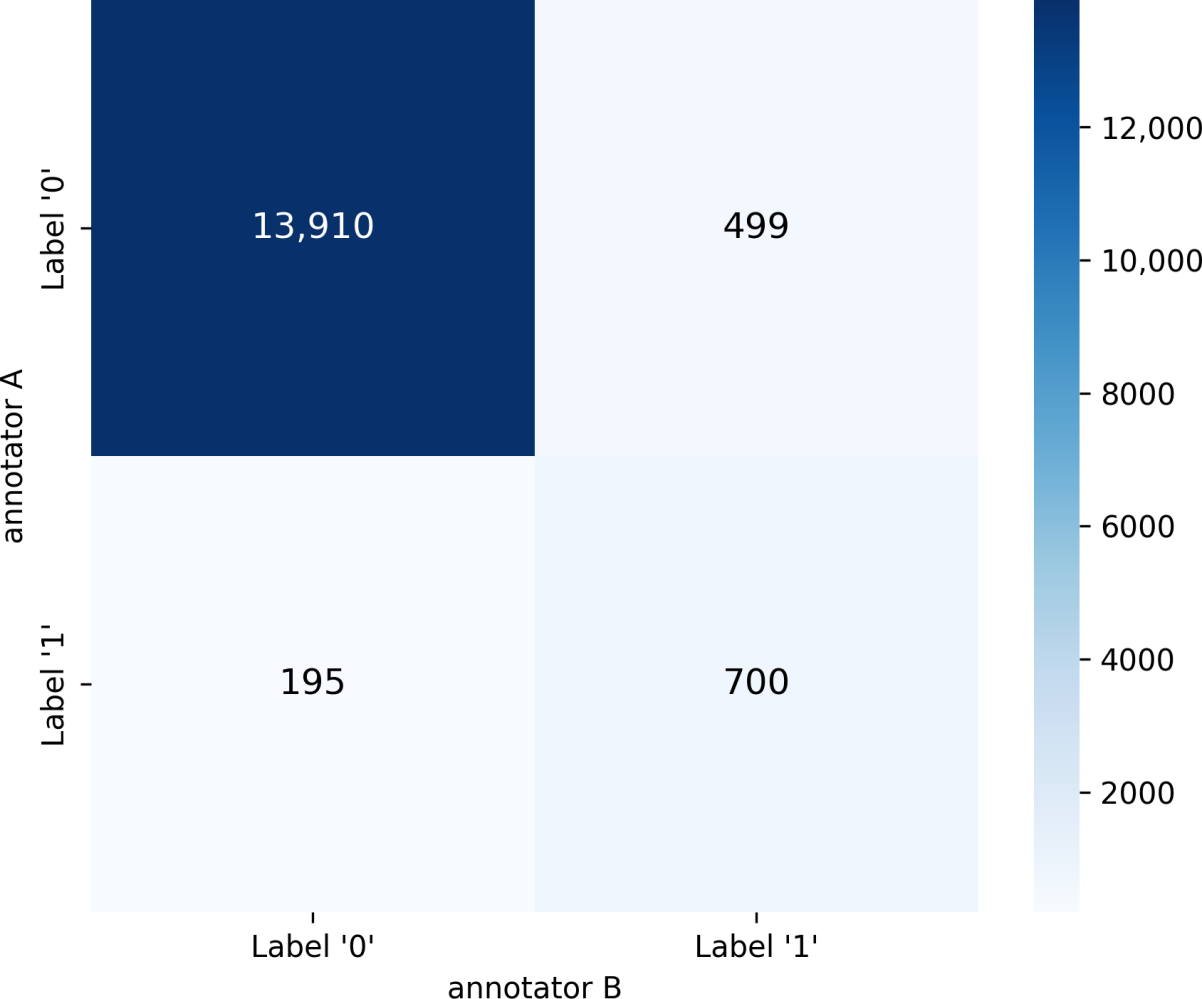
Confusion matrix showing interannotator agreement. Values represent the number of documents assigned to each label by annotator A and annotator B.

To quantitatively assess the quality and consistency of the manual annotation process, we computed interannotator agreement metrics and analyzed the final distribution of labels across the dataset. Following the labeling process described above, interannotator agreement was assessed across the full dataset of 15,304 documents. A total of 14,610 agreements and 694 disagreements were recorded between the 2 annotators. The distribution of label combinations was as follows: both annotators assigned label “0” in 13,910 cases and label “1” in 700 cases, while disagreements involved 499 (label “0” vs label “1”) and 195 (label “1” vs label “0”) instances. Based on this distribution, the Cohen κ coefficient was calculated, yielding a value of 0.645, which indicates substantial agreement and supports the reliability of the annotation process. The distribution of annotation combinations is summarized in Figure 2, which presents the confusion matrix of interannotator agreement.

All disagreements were adjudicated by a cardiology specialist in accordance with the predefined guidelines. Of the 694 discordant cases, 254 were assigned label “0” (absence of symptom) and 440 were assigned label “1” (presence of symptom). Following this resolution, the final distribution of labels in the dataset comprised 1140 documents labeled as “1” (7.5%) and 14,164 as “0” (92.5%), reflecting a high level of class imbalance that increases the complexity of the classification task.

### Experimentation Framework

The Transformer architecture has become a cornerstone in NLP, offering high efficiency and versatility across a wide range of tasks. Its ability to model long-range dependencies through attention mechanisms has revolutionized the field, driving significant advancements in tasks such as text classification [17-19]. This architecture is distinguished not only by its accuracy but also by its scalability and adaptability across diverse domains and datasets of varying sizes. Transfer learning allows for domain-specific adaptation through fine-tuning, as applied here to the health care domain [20]. By leveraging pretrained models that have already learned general language representations from large, diverse corpora, transfer learning facilitates their specialization for more domain-specific datasets through additional training. This adaptability has established Transformers as a powerful tool for advancing NLP in specialized fields.

A key limitation of Transformer models is their inherent constraint in processing large texts. Their architecture is typically designed to handle a maximum of 512 tokens, posing challenges for tasks that require longer sequences, such as document-level analysis or summarization of extensive texts. To address this limitation, the Longformer architecture [21], specifically designed to efficiently handle longer text sequences, was introduced. Longformer incorporates sparse attention mechanisms, allowing it to extend the context window while preserving computational efficiency, thus mitigating a core limitation of traditional Transformer models. This innovation broadens the applicability of Transformer models to tasks involving lengthy documents, such as EHRs. These considerations motivated the inclusion of 3 model categories in this study: general-purpose Transformers, domain-adapted clinical models, and Longformers. This categorization allows for a comprehensive comparison of performance across different levels of domain specialization and sequence-length flexibility. General-purpose models serve as a baseline, while fine-tuned clinical models assess the added value of domain-specific adaptation. Longformers, by contrast, address the limitations of standard sequence lengths and offer insight into the role of extended context modeling in clinical NLP.

In clinical contexts, maximizing recall for the positive class (Recall-1) is often prioritized to capture as many at-risk cases as possible. However, this strategy can lead to an increased number of false positives, elevating alert rates and potentially burdening health care systems. To address this trade-off, we incorporated additional evaluation metrics, specifically the AUC and the *F*_1_-score, to support a more balanced classification approach. Each metric provides a distinct perspective: AUC assesses the model’s overall ability to discriminate between positive and negative cases, while *F*_1_-score balances precision and sensitivity, helping to reduce both false positives and false negatives.

#### Models

As described in the previous section, this study evaluated 3 categories of encoder-based Transformer models: 2 general-purpose, 4 domain-adapted clinical models, and 2 Longformers. General-purpose models serve as a baseline to assess performance in the absence of domain adaptation, whereas fine-tuned clinical models illustrate the effects of transfer learning on domain-specific performance. Longformer models, designed to efficiently process extended text sequences, were included to address tasks requiring long-range contextual understanding, such as detailed medical reports or full-length patient narratives.

1. *BETO*. The Spanish-BERT model [22] follows the same architecture as BERT-Base and was trained exclusively on Spanish corpora, including Wikipedia and OPUS data. Specifically, we used the *bert-base-spanish-wwm-cased* model.
2. *RoBERTa*. A robustly optimized BERT pretraining approach, RoBERTa is an improved version of BERT that incorporates modifications to key hyperparameters [23]. Several Spanish-language RoBERTa models are available. We used the version pretrained on corpora from the National Library of Spain [24]. Specifically, experiments were conducted using the base version, *roberta-base-bne*.
3. *RoBERTa-biomedical* and *RoBERTa-biomedical-clinical* [25] are monolingual Spanish RoBERTa-based models trained on a large biomedical and clinical corpus of over 1 billion tokens. These models have demonstrated strong performance in prior Spanish-language biomedical and clinical NLP benchmarks. The specific versions used in this study were *roberta-base-biomedical-es* and *roberta-base-biomedical-clinical-es*.
4. *bsc-bio* and *bsc-bio-ehr* [26] are recently developed Spanish-language models, derived from *roberta-base-biomedical-es* and *roberta-base-biomedical-clinical-es*, respectively, and trained on expanded corpora to improve performance. Specifically, *bsc-bio* is a pretrained model for processing biomedical and clinical texts, suitable for tasks such as literature analysis, information extraction, and interpretation of medical guidelines. In contrast, *bsc-bio-ehr* is specifically adapted for processing EHRs and clinical notes.
5. *Long Transformer RoBERTa*. This model is a Longformer-based adaptation of RoBERTa for the Spanish language [24]. It combines sliding-window (local) and global attention mechanisms, enabling linear scalability with sequence length and facilitating the processing of documents with thousands of tokens. For this study, we used the model *longformer-base-4096-bne-es*.
6. *Long Transformer Biomedical-clinical*. This model extends the RoBERTa-based architecture through Longformer adaptations tailored for Spanish biomedical and clinical texts [26]. Initialized from the *roberta-base-biomedical-clinical-es* checkpoint, it underwent further fine-tuning using masked language modeling, specifically targeting long biomedical and clinical documents. We used the model *longformer-base-4096-biomedical-clinical-es* in our experiments. This model, explicitly designed for processing extended biomedical and clinical texts in Spanish, leverages domain-specific pretraining to achieve strong performance in NLP tasks within the health care domain, including the classification of clinical notes.

#### Evaluation Metrics

The models were evaluated using standard performance metrics commonly used in binary classification tasks for clinical documents. Specifically, the following metrics were considered.

##### Precision

Precision is the proportion of correctly predicted positive clinical documents relative to the total number of documents classified as positive. It is computed as:


(1) $$precision=\;\frac{true\;positives}{true\;positives\;+\;false\;positives}$$

##### Recall (Sensitivity)

Recall is the proportion of correctly predicted positive clinical documents relative to the total number of documents classified as positive. It is computed as:


(2) $$sensitivity=\;\frac{true\;positives}{true\;positives\;+\;false\;negatives}$$

##### *F*_1_-Score

*F*_1_-score is the harmonic mean of precision and recall, providing a single metric that balances both aspects of classification performance. It is computed as:


(3) $$F1-score=\;\frac{2\;*\;precision\;*\;recall}{precision\;+\;recall}$$

##### Specificity

Specificity is the proportion of correctly predicted negative clinical documents relative to the total number of actual negative documents. It is computed as:


(4) $$specifity=\;\frac{true\;negatives}{true\;negatives\;+\;false\;positives}$$

##### AUROC Metric

The AUROC is a performance metric used to evaluate binary classification models. It measures the model’s ability to distinguish between positive and negative classes, independent of the decision threshold. The ROC curve plots the true positive rate (or sensitivity) against the false positive rate (or 1-specificity) across different threshold values. For all metrics, a *positive document* indicates that it contains the symptom, while a *negative document* indicates that the symptom is not found within the text of the document.

### Experimental Methodology

#### Overview

Building robust models for detecting complex symptoms, such as those associated with HF in clinical contexts, requires fine-tuning of pretrained models and optimizing both hyperparameters and training strategies for the target task. To this end, the experimental framework was structured into two phases: (1) an initial hyperparameter search using a reduced dataset and (2) a subsequent full training and evaluation phase. During Phase 1, multiple hyperparameter configurations were systematically evaluated for each target metric, with the goal of optimizing models to maximize AUC, *F*_1_-score, or recall for the positive class. Phase 2 applied the optimal hyperparameter configurations identified previously to train each model on the full dataset and evaluate performance on the test dataset, enabling the selection of the best-performing configuration per strategy.

Regarding the preprocessing and normalization of the documents, all texts in the dataset were processed using the tokenizers associated with each pretrained model. No explicit preprocessing was applied in this study to preserve the original lexical and syntactic structure of the clinical narratives. This decision was motivated by the architecture of Transformer-based models, which are designed to capture contextual dependencies directly from raw, unprocessed text.

#### Phase 1: Hyperparameter Tuning

To limit computational demands, a further reduced subset of the training dataset was used during the hyperparameter search. This subset included 2000 randomly sampled instances obtained through undersampling, comprising 1500 Class “0” and 500 Class “1” examples. The reduction was performed in a proportionally weighted manner to preserve the original distribution of document types within the majority class. Table 4 summarizes the hyperparameter search space used in our experimentation.

A comprehensive grid search was conducted to evaluate all possible combinations of defined parameter values. This approach allows for systematic exploration of the hyperparameter space to identify the optimal configuration for each target performance metric. Optimization was carried out independently for each predefined strategy: (1) maximizing AUC, (2) maximizing *F*_1_-score, and (3) maximizing recall (sensitivity) for the positive class.

Tables 5-7 present the models and their optimal configurations for each optimization strategy, detailing key parameters such as batch size, learning rate, weight decay, and optimizer. This systematic, metric-driven optimization process enables the identification of the ideal configuration for each model based on the target metric, thereby enhancing model performance according to the specific requirements of the symptom classification task. As shown in these tables, the optimal hyperparameter values vary depending on the optimization strategy used. For instance, the bio model achieves better performance in AUC optimization when trained with a batch size of 16. However, its performance improves for *F*_1_-score and sensitivity optimization when batch sizes of 32 and 8 are used, respectively. By conducting an exhaustive search for these values, the final models attained optimal performance for each metric.

**Table 4.** Hyperparameter search space.

Hyperparameter	Values
Number of epochs	10 (using early stopping)
Batch size	[8, 16, 32]
Learning rate	[2e-5, 3e-5, 5e-5]
Weight decay	[0.1, 0.01, 0.001]
Optimizer	[adamw_hf, adamw_torch, adafactor]

**Table 5.** Best hyperparameter values for the area under the curve optimization strategy.

Model	Batch size	Learning rate	Weight decay	Optimizer
BETO	8	5e-05	0.001	adamw_torch
RoBERTa	8	2e-05	0.01	adafactor
RoBERTa-biomedical	16	2e-05	0.01	adamw_torch
RoBERTa-biomedical-clinical	16	5e-05	0.001	adamw_torch
bsc-bio	16	5e-05	0.001	adafactor
bsc-bio-ehr	32	3e-05	0.01	adamw_hf
Long Transformer RoBERTa	8	3e-05	0.01	adamw_hf
Long Transformer Biomedical-clinical	8	5e-05	0.01	adamw_hf

**Table 6.** Best hyperparameter values for the *F*_1_-score optimization strategy.

Model	Batch size	Learning rate	Weight decay	Optimizer
BETO	8	5e-05	0.001	adamw_torch
RoBERTa	16	5e-05	0.01	adafactor
RoBERTa-biomedical	16	5e-05	0.01	adamw_torch
RoBERTa-biomedical-clinical	8	3e-05	0.1	adamw_hf
bsc-bio	32	5e-05	0.1	adafactor
bsc-bio-ehr	8	2e-05	0.01	adamw_torch
Long Transformer RoBERTa	8	3e-05	0.01	adamw_hf
Long Transformer Biomedical-clinical	8	3e-05	0.001	adamw_hf

**Table 7.** Best hyperparameter values for the sensitivity optimization strategy.

Model	Batch size	Learning rate	Weight decay	Optimizer
BETO	8	5e-05	0.1	adamw_torch
RoBERTa	8	2e-05	0.01	adafactor
RoBERTa-biomedical	16	2e-05	0.001	adamw_torch
RoBERTa-biomedical-clinical	32	5e-05	0.001	adamw_hf
bsc-bio	8	5e-05	0.1	adamw_hf
bsc-bio-ehr	8	2e-05	0.1	adamw_hf
Long Transformer RoBERTa	8	2e-05	0.1	adamw_torch
Long Transformer Biomedical-clinical	8	5e-05	0.001	adafactor

#### Phase 2: Final Training and Evaluation

Using the optimal hyperparameters identified in Phase 1, the pretrained models were subsequently fine-tuned, each according to a specific optimization strategy (AUC, *F*_1_-score, or sensitivity). Given the pronounced class imbalance in the original training dataset, with a much larger proportion of majority class (label “0”) samples than minority class (label “1”), a reduced training dataset was created using undersampling. All minority class examples (n=1176) were retained, and 3 times that number were randomly sampled from the majority class (n=3528), yielding a training dataset of 4704 instances. To enable model tuning without compromising the final evaluation, 20% of the reduced training dataset was reserved as a validation dataset. Undersampling helps mitigate class imbalance by ensuring a more equitable representation of classes. This technique yields a manageable dataset size while maintaining a class distribution appropriate for the task, especially for detecting clinical symptoms in both classes. To preserve model generalizability, the test dataset was kept intact and fully isolated from the training process, enabling objective performance evaluation across the full dataset. Although undersampling can lead to the exclusion of some majority class instances, this trade-off was deemed acceptable to ensure an efficient and clinically relevant training process.

Alternative strategies to handle class imbalance were also considered during experimentation [27]. Oversampling methods, such as SMOTE (synthetic minority oversampling technique), were discarded due to their incompatibility with text-based data, as synthetic samples generated in vector space tend to lack semantic coherence. We also tested generating synthetic minority-class documents using pretrained language models, but the results were often clinically implausible or grammatically inconsistent. Additionally, class weighting was implemented in early trials, but this approach led to unstable training dynamics and overfitting to the minority class, particularly in long-document models. Based on these observations, stratified undersampling was selected as the most effective and interpretable solution for our setting.

## Results

This section presents and analyzes the results obtained on the test dataset for the models trained under the 3 previously described optimization strategies. The dataset was divided into training (80%), validation (20%), and test (20%) subsets to support model development and evaluation. Table 8 shows the number of documents in each dataset partition. As previously described, an undersampling strategy was applied to the training and validation datasets to mitigate class imbalance.

**Table 8.** Size and label distribution in the training, validation, and test datasets.

Dataset	Number of documents	Label “0”	Label “1”
Original	15,304	13,853	1451
Training	3764	2823	941
Validation	940	705	235
Test	3032	2757	275

To improve model robustness and ensure greater statistical validity, two strategies were implemented in the construction of the test dataset:

1. Ensuring patient-level separation between the training and test datasets. That is, test documents originated from patients not included during model training. This ensured that the test dataset contained entirely unseen data for the model. Satisfactory results under this condition would indicate strong generalization capability in detecting HFpEF symptoms.
2. Preserving the original class distribution in the test dataset (91% class “0,” 9% class “1”). This strategy allows evaluation under realistic conditions reflecting the natural class imbalance between negative and positive HFpEF cases.

**Figure 3.**
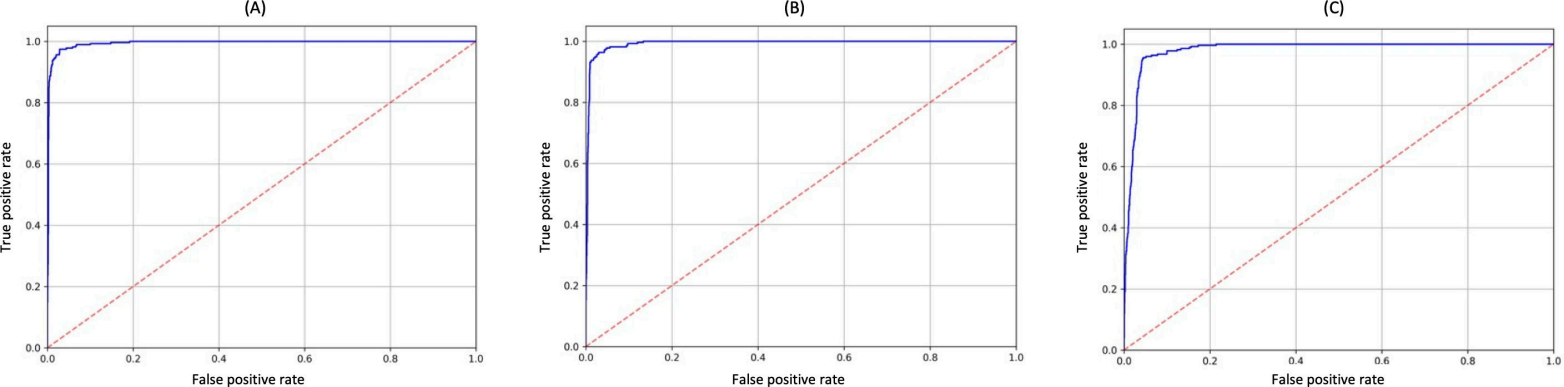
ROC curves for the test dataset, illustrating the performance of models optimized using 3 different hyperparameter optimization strategies. (**A**) Optimization based on AUC using the *Longformer Biomedical-clinical* model; (**B**) Optimization based on *F*_1_-score using the same model; (**C**) Optimization based on sensitivity using the *bsc-bio-ehr* model. For each strategy, the model shown corresponds to the one that achieved the highest AUC on the test dataset.AUC: area under the curve; ROC: receiver operating characteristic.

Tables9^11^ summarize the performance of the 6 selected language models trained under the 3 optimization strategies. The evaluation metrics indicate robust and consistent performance across models. A key observation is the consistently high AUC values across models, all surpassing the 0.80 threshold considered clinically significant [28]. Figure 3 displays the ROC curves of the models that achieved the best performance in terms of AUC.

Overall, the *Long Transformer Biomedical-clinical* model achieved the highest performance, highlighting that domain-adapted pretrained models generally outperform general-purpose counterparts in specialized clinical tasks. Moreover, models capable of processing extended input sequences exhibit superior suitability for clinical domains, where documents often exceed the standard 512-token limitation of conventional Transformer architectures.

**Figure 4.**
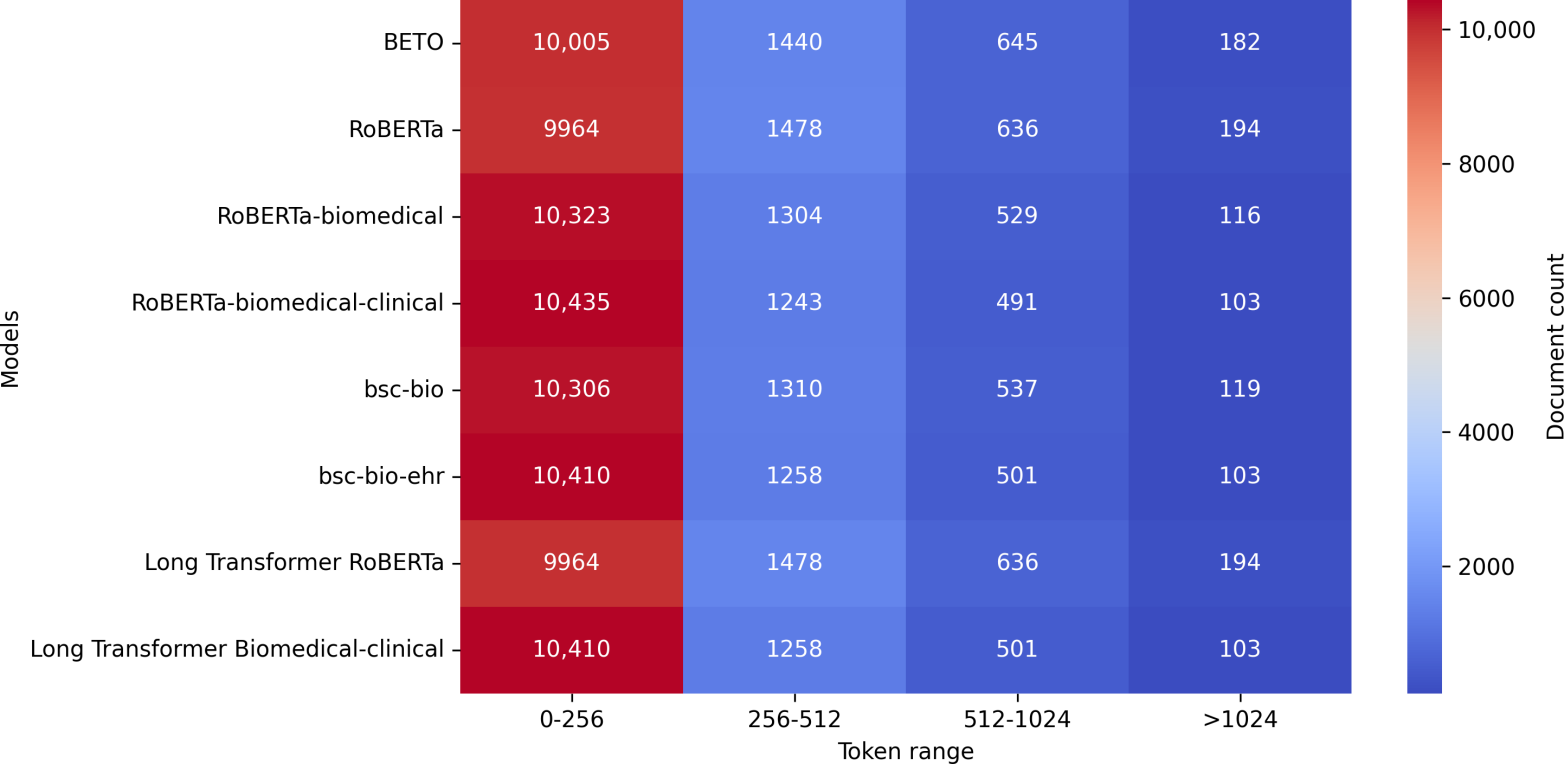
Distribution of document lengths (in tokens) across the full dataset, computed using each model’s tokenizer. The x-axis represents token count ranges, and the y-axis lists the tokenizers associated with the evaluated language models.

As shown in Figure 4, analysis of token distributions across models revealed that the majority of clinical documents (approximately 85%‐95%, depending on the tokenizer) contain 512 tokens or fewer. However, a nonnegligible subset of documents (5%‐15%) exceeds this limit, underscoring the presence of longer narratives in the dataset. In standard Transformer-based models, these longer documents were truncated during processing due to sequence length limitations. Although this limitation may result in the loss of relevant contextual information, the models still achieved robust performance, indicating their effectiveness even when operating on truncated inputs. To better handle longer documents, Longformer-based models were used, enabling a maximum input length of 1024 tokens. This extended capacity allows for more complete document representations while preserving computational efficiency. Although only a small fraction of the dataset (1%‐2%) exceeded 1024 tokens, the increased sequence length enhanced contextual coverage for longer documents compared to standard Transformer models. The inclusion of Longformers in the experimental framework highlights their value in handling tasks involving lengthy and complex texts, particularly in clinical NLP, where capturing contextual nuances is crucial for effective classification.

**Figure 5.**
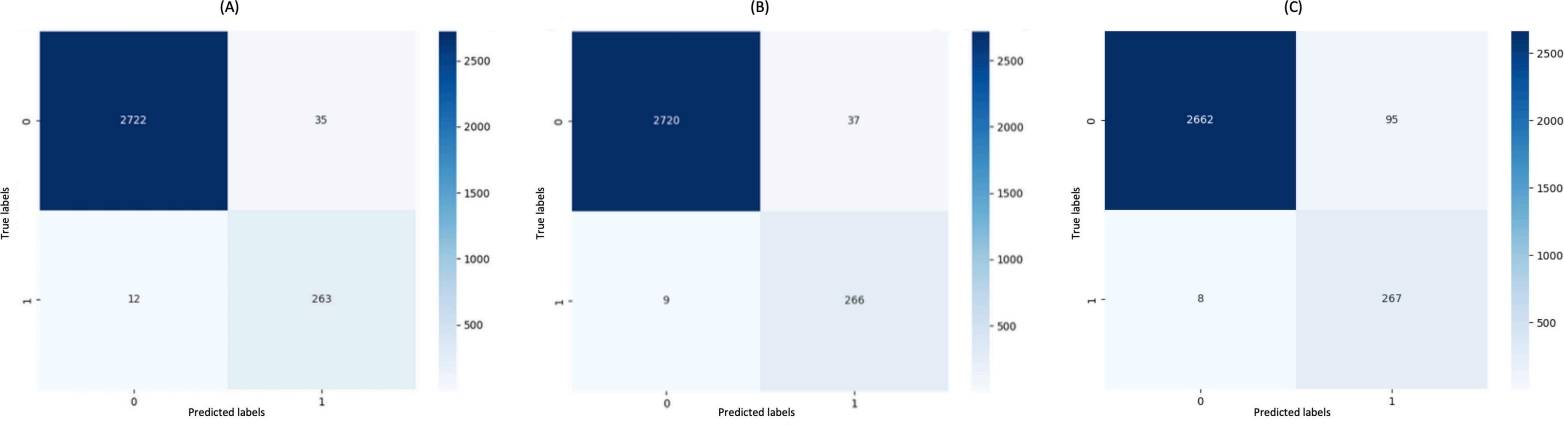
Confusion matrices showing classification results on the test dataset using 3 different hyperparameter optimization strategies: (**A**) AUC-based optimization using the *Longformer Biomedical-Clinical* model; (**B**) *F*_1_-score–based optimization using the same model; and (**C**) sensitivity-based optimization using the *Longformer RoBERTa* model. Each matrix shows the number of true positives, true negatives, false positives, and false negatives predicted by the respective model. These results illustrate the trade-offs introduced by each optimization criterion in terms of sensitivity and specificity. AUC: area under the curve.

In diagnostic applications, minimizing the false negative rate is critical to improving the model’s ability to correctly identify positive cases and facilitate early disease detection. Accordingly, sensitivity was prioritized in one experimental condition by training a model specifically optimized for this metric. This approach increased the correct classification rate of positive cases, thereby reducing the likelihood of missing patients in need of further clinical evaluation. To evaluate the impact of each optimization strategy on model performance, confusion matrices were analyzed on the test dataset. Figure 5 shows the confusion matrices of the best-performing models under each optimization strategy.

The trained Transformer-based models exhibited high sensitivity across all 3 optimization strategies, consistently achieving low false-negative rates. In particular, classification accuracy for the positive class exceeded 96% in all cases, highlighting the robustness of both the annotated corpus and the experimental design. These findings underscore not only the models’ effectiveness in detecting positive cases, but also the consistency and reliability of the adopted methodology. Confusion matrix analysis reveals subtle performance differences depending on the optimization strategy applied. Notably, the sensitivity-optimized strategy reduced false negatives to just 8, resulting in a 97% accuracy rate for positive case detection. However, this improvement came at the cost of increased false positives (95), illustrating the inherent trade-off between sensitivity and specificity. In contrast, the AUC and *F*_1_-score strategies, which aim to balance accuracy across classes, significantly reduced false positives (35 and 37, respectively). Simultaneously, these strategies maintained relatively low false-negative counts (12 and 9, respectively).

**Table 9.** Performance metrics obtained on the test dataset for all evaluated models under the AUC^a^-based optimization strategy.

Model	Sensitivity	Specificity	*F*_1_-score	AUC
BETO	0.920	0.960	0.959	0.940
RoBERTa	0.938	0.985	0.983	0.962
RoBERTa-biomedical	0.960	0.972	0.972	0.966
RoBERTa-biomedical-clinical	0.967	0.961	0.964	0.964
bsc-bio	0.945	0.980	0.977	0.963
bsc-bio-ehr	0.953	0.980	0.978	0.966
Long Transformer RoBERTa	0.960	0.952	0.957	0.956
Long Transformer Biomedical-clinical	0.956	0.987	0.985	0.971

a AUC: area under the curve.

**Table 10.** Performance metrics obtained on the test dataset for all evaluated models under the *F*_1_-score–based optimization strategy.

Model	Sensitivity	Specificity	*F*_1_-score	AUC
BETO	0.949	0.935	0.942	0.942
RoBERTa	0.967	0.946	0.952	0.957
RoBERTa-biomedical	0.985	0.849	0.885	0.917
RoBERTa-biomedical-clinical	0.967	0.923	0.936	0.945
bsc-bio	0.956	0.971	0.971	0.963
bsc-bio-ehr	0.956	0.971	0.971	0.964
Long Transformer RoBERTa	0.920	0.987	0.981	0.954
Long Transformer Biomedical-clinical	0.987	0.987	0.985	0.987

**Table 11.** Performance metrics obtained on the test dataset for all evaluated models under the sensitivity-based optimization strategy.

Model	Sensitivity	Specificity	*F*_1_-score	AUC^a^
BETO	0.938	0.955	0.957	0.947
RoBERTa	0.938	0.986	0.983	0.962
RoBERTa-biomedical	0.953	0.934	0.942	0.943
RoBERTa-biomedical-clinical	0.967	0.925	0.937	0.946
bsc-bio	0.985	0.943	0.952	0.964
bsc-bio-ehr	0.960	0.978	0.977	0.969
Long Transformer RoBERTa	0.979	0.956	0.968	0.968
Long Transformer Biomedical-clinical	0.971	0.922	0.935	0.946

a AUC: area under the curve.

## Discussion

### Principal Results

This study shows that the performance of Transformer-based models in HFpEF symptom detection can be substantially influenced by the chosen optimization strategy. Each approach targeting (AUC, *F*_1_-score, or sensitivity) serves a distinct clinical and operational objective. Notably, the sensitivity-driven strategy proved particularly effective in minimizing false negatives, a critical consideration in early disease detection scenarios. This highlights the value of aligning model training objectives with specific clinical priorities when developing NLP tools for medical applications.

It should be noted that direct comparisons with related studies are not feasible, as each uses different datasets. Nevertheless, the strength of the results confirms the validity of the experimental design and the robustness of the trained models under the proposed optimization strategies, as reflected in the evaluation metrics. Additionally, the involvement of cardiology specialists provides expert validation and qualitative support for these findings.

Beyond retrospective evaluation, two real-world integration scenarios are envisioned: (1) batch-mode screening, where the model is periodically applied to existing EHR data to prioritize patients for further testing, and (2) real-time alerting, where symptom mentions in clinical notes trigger nonbinding alerts to the clinician. In either case, the system would function as a clinical decision support tool. To mitigate false positives, adjustable probability thresholds and explanatory interfaces that highlight the relevant text segments are recommended. These strategies align with current frameworks for trustworthy and transparent AI in health care.

### Error Analysis

A qualitative error analysis was performed from a clinical perspective, focusing on both false negative and false positive cases. Special attention was given to false negatives to identify the reasons why the models predicted the absence of the symptom in a document (label “0”) when, in reality, it had been classified as positive, meaning that the document contained information indicating the presence of the symptom. We selected the misclassified documents from the 3 models with the lowest false negative rates (see Figure 5).

Textbox 1 presents a false negative example, in which all models predicted the case as negative, despite human annotation indicating the presence of the symptom. However, a review of the annotation guidelines revealed that the annotator’s interpretation relied on clinical reasoning that, while valid, extended beyond the defined annotation criteria. The text includes terms such as *“cardiomegalia, signos de hipertensión pulmonar precapilar y poscapilar con infiltrados de edema intersticial alveolar bilaterales”* (“cardiomegaly, signs of pre- and post-capillary pulmonary hypertension with bilateral interstitial-alveolar edema infiltrates”), which can be associated with HFpEF. However, the guidelines did not explicitly state that these findings alone were sufficient for a positive classification without additional diagnostic information or clinical context. In this regard, the model’s prediction strictly adhered to the established criteria, whereas the human annotation reflected a broader interpretation based on implicit clinical knowledge. This example highlights the inherent complexity of symptom detection and the subtle distinction between rule-based annotation and clinically contextual judgment. Despite these occasional discrepancies, the models have demonstrated a high level of consistency in applying the predefined criteria. Future revisions of the annotation process could explore potential adjustments to better align guideline application with the expected clinical reasoning for this task.

Conversely, Textbox 2 shows an example of a false positive error. In this case, the models incorrectly predicted the presence of HFpEF symptoms in a document explicitly indicating their absence. Although the key phrase *“no existe daño renal ni signos de ICC”* (“no kidney damage or signs of CHF”) clearly indicated the negative class, the models failed to interpret the negation correctly in this context. Such errors are a well-known challenge in clinical NLP, where negation is often expressed using diverse and context-dependent linguistic patterns. Notably, all 3 models produced the same misclassification, indicating that the difficulty in interpreting negation structures is model-independent.

Despite these specific instances, the overall performance of the models has been consistent across most evaluated cases. However, future improvements could focus on refining negation handling through domain-adapted preprocessing pipelines or model adaptations that explicitly account for linguistic cues. In particular, the integration of rule-based algorithms, enhanced tokenization schemes, or syntactic features could support more accurate interpretation of negated or speculative statements. These enhancements represent promising avenues for improving model robustness in real-world clinical applications.

Textbox 1. **Example of a misclassified document (false negative). Document manually labeled as positive and classified as negative by all three classifiers. The English translation is included to facilitate reading.**

**Spanish:**

*Ecg: en fa a unos 135-140 lpm, e isquemia subendocardica lateral.cr 1.6, probnp 6869, tnt 20, iones normales, hb 10.7g, inr tp 2.8.bq planta: cr 1.40mg, ggtp 84u, urea 68mg, iones normales, hierro 24 mcg, ab 3.3g, mg 1.52 mg.pco2 63, ph 7.33, bicarbonato 33.orina normal.rx de torax: cardiomegalia, signos de hipertension pulmonar precapilar y poscapilar con infiltrados de edema intersticioalveolar bilaterales.*

**English:**

ECG: atrial fibrillation at approximately 135-140 bpm, with lateral subendocardial ischemia. CR 1.6, ProBNP 6869, TnT 20, normal ions, Hb 10.7 g, INR/TP 2.8. Ward: CR 1.40 mg, GGTP 84 U, urea 68 mg, normal ions, iron 24 mcg, AB 3.3 g, Mg 1.52 mg. PCO2 63, pH 7.33, bicarbonate 33. Urinalysis: normal. Chest X-ray: cardiomegaly, signs of pre- and postcapillary pulmonary hypertension, with bilateral interstitial-alveolar edema infiltrates.

Textbox 2. **Example of a misclassified document (false positive). Document manually labeled as negative and classified as positive by all 3 classifiers. The English translation is included to facilitate reading.**

**Spanish:**

*Hoy tiene prevista tercera infusión del fármaco. peso 49.5 kg según lo recogido hoy. clínicamente bien, salvo edemas en ambos maléolos, creo que en relación al patisiran pues no existe daño renal ni signos de icc.ef. beg. acr con cor rítmico, sin soplos audibles, con bmv. abdomen sin masas ni megalias. mmii cion edemas que dejan fóve a nivel maleolar. no signos de tvp.plan:- próxima cita 10 de julio- controles analíticos los de nefrología por ahora (niveles de tacrolimus en rango en último control).*

**English:**

Today, the third infusion of the drug is scheduled. Weight: 49.5 kg as recorded today. Clinically well, except for edema in both malleoli, which I believe is related to patisiran, as there is no renal damage or signs of heart failure. Physical examination: well-nourished and in good general condition. Peripheral circulation: rhythmic heart sounds, no audible murmurs, with good bilateral breath sounds. Abdomen: no masses or organ enlargement. Lower limbs: with edema that leaves a pit at the malleolar level. No signs of deep vein thrombosis. Plan: next appointment: July 10. Laboratory tests: nephrology monitoring for now (tacrolimus levels within range in the last test).

### Clinical Implications of Misclassification Errors

From a clinical standpoint, accurately identifying patients with suspected HFpEF is critical for ensuring appropriate management. Classification errors may result in the omission of key diagnostic procedures or delays in initiating disease-modifying therapies. In particular, false negatives represent a major concern, as they may leave early-stage patients undiagnosed and untreated. Conversely, false positives may prompt unnecessary diagnostic procedures, increasing both clinical workload and health care costs. Therefore, tuning strategies that prioritize sensitivity while preserving acceptable specificity are essential for the effective deployment of these models in clinical settings.

### Limitations

This study has several limitations that should be acknowledged. First, all clinical data were obtained from a single hospital in Spain, using one specific EHR system. As a result, the trained models reflect documentation practices, linguistic patterns, and institutional conventions specific to that context. This may limit the generalizability of the findings to other health care settings, regions, or EHR platforms. Second, no external validation was performed on datasets from other institutions or countries. Although the models demonstrated strong internal performance, further evaluation on external corpora will be essential to assess their robustness and adaptability to different clinical environments and documentation styles. As highlighted in recent studies, external validation is a critical step to ensure the reliability and applicability of NLP models across diverse health care systems [29]. Despite these limitations, this work provides a valuable foundation for developing NLP systems tailored to Spanish-language clinical narratives and highlights the feasibility of symptom detection in the context of cardiac amyloidosis. Future research should focus on validating and refining these models in multicenter, cross-national settings to enhance their clinical relevance.

### Discussion of Ethical Considerations

This study was conducted using anonymized clinical data in accordance with the General Data Protection Regulation (regulation [EU] 2016/679) and Spanish data protection laws (*Ley Orgánica 3/2018 de Protección de Datos Personales y garantía de los derechos digitales*). Data use was approved by the institutional ethics committee. Looking forward, any clinical deployment of the proposed AI system would need to comply with the EU Artificial Intelligence Act and, depending on its intended function, may also fall under the scope of the Medical Device Regulation (regulation [EU] 2017/745). Consequently, future implementations will require robust validation, human oversight mechanisms, and explainability features to ensure safe and ethical integration into clinical practice.

### Conclusions

This study proposed an experimental framework for optimizing Transformer-based language models to automatically detect clinical documents suggesting the presence of HFpEF. Our findings underscore the importance of hyperparameter tuning, as optimal configurations directly influence model performance according to the prioritized evaluation metric. Specifically, to minimize false negatives, optimization should prioritize model sensitivity. This approach allows model performance to be adapted to varying clinical and operational requirements, maintaining a suitable balance between sensitivity and specificity depending on the context of use.

It has been demonstrated that using of domain-specific pretrained language models significantly improves adaptability and transferability for specialized clinical tasks. This advantage lies in the ability of context-aware models to better capture linguistic patterns, domain-specific terminology, and semantic relationships, thereby enhancing task-specific performance. Moreover, the capacity to process longer sequences during fine-tuning and inference is especially beneficial in the clinical domain. Medical documents are often lengthy and rich in interrelated concepts, which may be lost when contextual processing is constrained. Therefore, using models designed for extended input sequences is essential for comprehensive and accurate clinical text analysis.

Having validated our experimental approach and confirmed model reliability for HFpEF detection, future work will focus on developing and fine-tuning models capable of comprehensively identifying all symptoms associated with cardiac amyloidosis. To this end, the top-performing models from this study will be retrained on manually annotated corpora to incorporate the full range of clinically relevant manifestations. This process will improve the system’s ability to detect textual patterns indicative of the disease with increased precision and sensitivity. The ultimate aim is to implement an automated screening system based on EHR analysis to support the early identification of patients with suspected cardiac amyloidosis. This approach may optimize diagnostic workflows, improve clinical decision-making, and enable more personalized treatment strategies for a condition that remains difficult to detect at early stages.

## Supplementary material

10.2196/76433 Multimedia Appendix 1 Supplemental materials.

## Abbreviations

AI: artificial intelligence
ATTR-CM: transthyretin cardiac amyloidosis
AUC: area under the curve
AUROC: area under the receiver operating characteristic curve
EHR: electronic health record
HF: heart failure
HFpEF: heart failure with preserved ejection fraction
NLP: natural language processing
ROC: receiver operating characteristic
RQ: research question
