# Correction: Multicriteria Optimization of Language Models for Heart Failure With Preserved Ejection Fraction Symptom Detection in Spanish Electronic Health Records: Comparative Modeling Study

**DOI:** 10.2196/97621

**Published:** 2026-05-28

**Authors:** Jacinto Mata, Victoria Pachón, Ana Manovel, Manuel J Maña, Manuel de la Villa

**Affiliations:** 1 I²C Research Group, Universidad de Huelva, Huelva, 21007, Spain, +34 687862089; 2 Cardiology Department, Juan Ramón Jiménez University Hospital, Multidisciplinary Amyloidosis Unit Huelva, Hospital Juan Ramón Jiménez, Huelva, Spain

In “Multicriteria Optimization of Language Models for Heart Failure With Preserved Ejection Fraction Symptom Detection in Spanish Electronic Health Records: Comparative Modeling Study” [1], the authors made one correction.

In the Acknowledgments section, the following text was revised:

This project was funded by the Institute of Health Carlos III, Ministry of Science, Innovation and Universities, Spanish Government (grant number PI20/01485).

The text now reads:

This study has been funded by the Instituto de Salud Carlos III through the project PI20/01485 (cofunded by the European Regional Development Fund/European Social Fund “A way to make Europe”/“Investing in your future”).

The correction will appear in the online version of the paper on the JMIR Publications website, together with the publication of this correction notice. Because this was made after submission to PubMed, PubMed Central, and other full-text repositories, the corrected article has also been resubmitted to those repositories.
