# Supplementary material for: Multicriteria Optimization of Language Models for Heart Failure With Preserved Ejection Fraction Symptom Detection in Spanish Electronic Health Records: Comparative Modeling Study
Source: J Med Internet Res. 2025 Jul 17;27:e76433. doi: 10.2196/76433 (PMC12288768; doi:10.2196/76433)
Supplement: Multimedia Appendix 1 [file jmir-v27-e76433-s001.pdf]

Appendix

Sample of documents extracted from the dataset. (Automatic English translation is included to facilitate reading)

**Document**	**Label**
Consulta por urgencias hace 48hrs por sensación de palpitaciones y disnea en reposo así como tos escasamente productiva de una semana de evolución. Refiere dolor torácico atípico tipo pleurítico que aumenta con la inspiracion. Afebril hasta el momento. No otra sintomatología por aparatos ni sistemas. -Tabaquismo activo de 5 cig/día. Impresiona de mala adherencia al tratamiento y cumplimiento errático. En urgencias se describe con buen estado general. Hallazgo de acidosis respiratoria en GSV (pH: 7.27 y pCO2: 88 mmHg), que empeora tras la administración de aerosoloes, por lo que pasa a observación y se inicia soporte VNI, manteniéndose dependiente de la VNI por trabajo respiratorio y empeoramiento d pa hipercapnia tras su intento de retirada.	0
Emergency consultation 48 hours ago due to a sensation of palpitations and dyspnea at rest, as well as a mildly productive cough lasting one week. Reports atypical pleuritic chest pain that worsens with inspiration. Afebrile so far. No other symptoms affecting other systems. Active smoker, 5 cigarettes/day. Appears to have poor adherence to treatment and erratic compliance. In the emergency department, described as being in good general condition. Finding of respiratory acidosis in arterial blood gas (pH: 7.27 and pCO2: 88 mmHg), which worsened after aerosol administration, leading to observation and initiation of non-invasive ventilation (NIV), remaining dependent on NIV due to respiratory effort and worsening hypercapnia after attempted withdrawal.	0
Mujer de 78 años refiere aumento de disnea progresivo desde hace 2 meses, acentuado en los ultimos 5 días, NYHA III. Opresión torácica sin contejo vegetativo tanto en esfuerzo como con algunos episodios de reposo. Refiere ortopnea con episodios de DPN. Palpitaciones. Refiere edemas ocasionales en MMII. Refiere episodios de mareos frecuentes con caídas en relación con su cervicopatía y otros nocturnos de perfil ortostático sin síncopes. En ecg realizado en MAP, se observa TSV a 120 lmp, , En Urgencias, ecg en ritmo sinusal a 80 lpm. Se realiza placa de tórax con signos sugestivos de descompensación de origen cardiaco (pulmón congestivo, con signos de liquido interticial en cisura y borramiento senos costofrénicos), con Pro BNP 2500 y Hb 7, 8 . Se procede al sondaje vesical de la paciente y se administra 1 ampolla de furosemida . Al llegar a planta, se realiza trasfusión de 1 Concentrado de hematíes + Ampolla de fusosemida.	1

A 78-year-old woman reports a progressive increase in dyspnea over the past two months, worsening in the last five days, classified as NYHA III. She experiences chest tightness without autonomic symptoms, both with exertion and during some episodes at rest. Reports orthopnea with episodes of paroxysmal nocturnal dyspnea (PND). Palpitations. Occasional lower limb edema. Frequent dizziness episodes with falls related to her cervical pathology, as well as nocturnal episodes with an orthostatic profile, without syncope. An ECG performed by the primary care physician (PCP) shows supraventricular tachycardia (SVT) at 120 bpm. In the emergency department, the ECG shows sinus rhythm at 80 bpm. A chest X-ray reveals signs suggestive of cardiac decompensation (congestive lungs, interstitial fluid in the fissure, and blurring of the costophrenic angles), with Pro BNP of 2500 and hemoglobin (Hb) of 7.8. A urinary catheter is placed, and one ampoule of furosemide is administered. Upon admission to the ward, the patient receives a transfusion of one unit of packed red blood cells plus another ampoule of furosemide.	1
